# Race/Ethnicity, Enrichment/Fortification, and Dietary Supplementation in the U.S. Population, NHANES 2009–2012

**DOI:** 10.3390/nu11051005

**Published:** 2019-05-02

**Authors:** Angela M. Malek, Jill C. Newman, Kelly J. Hunt, Bernadette P. Marriott

**Affiliations:** 1 Department of Public Health Sciences, Medical University of South Carolina, Charleston, SC 29425, USA; huntke@musc.edu; 2 Department of Medicine, Medical University of South Carolina, Charleston, SC 29425, USA; newmanji@musc.edu; 3 Departments of Medicine and Psychiatry, Medical University of South Carolina, Charleston, SC 29425, USA; marriobp@musc.edu

**Keywords:** dietary reference intake, socioeconomic status

## Abstract

In the United States (U.S.), food fortification and/or enrichment and dietary supplement (DS) use impacts nutrient intakes. Our aim was to examine race/ethnicity and income (Poverty Income Ratio, PIR) differences in meeting the Dietary Reference Intakes based on estimated dietary intakes among the U.S. population age ≥2 years (*n* = 16,975). Two 24-hour recalls from the National Health and Nutrition Examination Survey (NHANES) cycles 2009–2012 were used to estimate the intake of 15 nutrients as naturally occurring, enriched/fortified, and plus DSs. Across racial/ethnic groups and within PIR categories, significant differences were observed in the %< Estimated Average Requirement (EAR) for vitamin A following enrichment/fortification (E/F) and for vitamin B_12_ and riboflavin following both E/F and DS use when comparing non-Hispanic blacks, Hispanics, and the other race/ethnicity group to non-Hispanic whites. The %<EAR for iron and calcium also differed depending on race/ethnicity within PIR category (*p* < 0.05). The %<EAR was significantly lower for vitamin D after E/F for Hispanics, and after E/F combined with DS use for vitamins C and B_6_ for Hispanics and the other race/ethnicity group than non-Hispanic whites. Non-Hispanic blacks were inadequate in all nutrients examined except vitamin C based on the %<EAR than individuals of other races/ethnicities. Differences in the tolerable upper intake level (UL) of nutrients, especially folate and zinc, also varied by race/ethnicity and PIR category.

## 1. Introduction

The United States (U.S.) Dietary Guidelines for Americans (DGA) 2015 recommended healthy eating patterns across the lifespan. However, many Americans (42.2%) do not adhere to these guidelines, according to the 2010 Healthy Eating Index (HEI) scores [1]. Furthermore, the 2015 Dietary Guidelines Advisory Committee (DGAC) identified a number of shortfall nutrients (folate, calcium, magnesium, fiber, potassium, and vitamins A, D, E, and C) among which consumption had not met the Institute of Medicine’s Dietary Reference Intakes (DRIs) of the Estimated Average Requirement (EAR) or the Adequate Intake (AI) [2]. While vegetables, fruits, whole grains, and dairy are important sources of shortfall nutrients, intake is low for many Americans [2]. Enrichment and/or fortification (E/F) of foods in the U.S. food supply has helped with meeting the recommended nutrient intake levels as well as reducing some deficiencies through replacement of specific nutrients lost during processing (enrichment) and increasing nutrient levels (fortification) [3,4]. The use of dietary supplements (DSs) has also played a role in meeting nutrient intake recommendations and reducing the percentage of the population that is below the EAR [5].

Racial/ethnic differences in shortfall nutrients were noted in the 2015 DGAC report (Part D), with a few notable trends. Intake of most nutrients was lowest among non-Hispanic blacks (NHB), vitamin C intake was highest for Hispanics, and intake of several other nutrients were highest among non-Hispanic whites (NHW) such as magnesium, folate, iron, potassium, calcium, and vitamins A, E, and D [2]. This disparity among the population is of concern given the associations between health outcomes and the under-consumed shortfall nutrients including calcium, potassium, vitamin D, and iron (among adolescent and premenopausal adult females) [2]. 

Disparities in adherence to the dietary recommendations by race/ethnicity and income among U.S. adults in general or with regard to a particular food group or food have also been examined [6]. In 2011, Wang and Chen observed lower HEI scores among NHB adults compared with NHW adults after adjustment for socioeconomic status (SES) and certain nutrition and health related psychosocial factors [6]. Previous research has assessed recommended intakes by socioeconomic level, such as Supplemental Nutrition Assistance Program (SNAP) eligibility. However, these studies have often been limited to a nutrient, food, or food group. Some studies have assessed usual intakes of many nutrients by the poverty index ratio (PIR) [7,8,9] or racial/ethnic differences [9,10]. However, only a few studies to date have included E/F and these studies have not focused on racial/ethnic differences [5,11,12]. 

Fulgoni et al. evaluated the total usual nutrient intakes for 19 micronutrients from naturally occurring, enriched/fortified, and DS sources (aged ≥2 years), using the North American branch of the International Life Sciences Institute (ILSI North America) Fortification database and the National Health and Nutrition Examination Survey (NHANES) 2003–2006 [5]. Results from this study showed that many Americans did not meet the DRI recommended micronutrient intake levels prior to E/F and DS [5]. Following E/F, intakes of vitamins A, C, and D, thiamin, iron, and folate dramatically improved, and further improved with the use of DSs [5]. Additionally, Berner et al. examined the impact of E/F on nutrient intakes among children and adolescents using the NHANES 2003–2006 [11], as well as intakes of certain nutrients with and without fortification by gender and age groups among children and adults aged ≥1 year using the Continuing Survey of Food Intakes by Individuals (CSFII) 1989–1991 dietary data [12].

Analyzing NHANES 2009–2012, Blumberg et al. (2017) recently found less apparent nutrient inadequacies among NHW adults than those in other racial/ethnic groups examined (NHB, Hispanic, and non-Hispanic Asian [NHA]) who reported using DSs [10]. Blumberg et al. (2017) also observed nutrient inadequacies that varied by PIR category [7]. Adults in the highest PIR category who reported using DSs experienced lower rates of inadequacy in twice as many nutrients compared to counterparts in the middle and lowest PIR categories [7].

Usual nutrient intakes of the eight shortfall nutrients among adults 19 years of age and older by PIR and sex using one NHANES cycle, 2011–2012, and two 24-hour dietary recalls were evaluated by Bailey et al. [8]. The authors found that, regardless of PIR category, men had significantly greater mean intakes of folate and vitamins C and D, whereas intake of magnesium was higher for women [8]. Compared with the lowest and middle PIR categories, the %<EAR for all micronutrients was significantly lower for those in the highest PIR category (PIR ≥ 350%), and mean total usual nutrient intakes were significantly higher for 7 of the 8 micronutrients in men (except calcium) and women (except vitamin C) [8].

Use of DSs including multivitamins and multi-minerals by the U.S. population ≥1 years of age has also been evaluated by SES and health-related characteristics with the NHANES 2003–2006 [13]. Self-reported DS use was highest for NHWs (59%) followed by 36% for NHBs and 34% for Mexican-Americans [13].

The aim of the current study is to determine if nutrient intakes differ among racial/ethnic groups in the U.S. population in individuals ≥2 years of age, when accounting for PIR, using NHANES dietary data for two cycles, 2009–2012, and the ILSI North America Fortification database. To achieve our aim, estimations of usual dietary intake based on the National Cancer Institute (NCI) usual intake estimation methodology [14,15] were used to compare intakes of 15 nutrients as they occur intrinsically in food, after E/F, and after the addition of DSs to intakes from enriched/fortified foods with the appropriate DRIs.

## 2. Materials and Methods

### 2.1. Study Population and Dietary Data

The NHANES is a nationally representative, cross-sectional survey conducted by the National Center for Health Statistics (NCHS) that uses a complex, stratified, multistage probability cluster sampling design to sample noninstitutionalized, civilian U.S. residents [16]. Approval for the NHANES protocol was received from the NCHS Research Review Board, and participants or proxies provided informed consent. Two 24-hour dietary recalls were collected as part of the NHANES using the computer-assisted dietary interview software program, USDA’s automated multiple-pass method (AMPM), and are available in the Centers for Disease Control and Prevention (CDC) public dietary data. These 24-hour dietary recalls include an in-person interview followed by a telephone interview administered three to 10 days later. In addition, DSs data are available through NHANES from the first day dietary interview during which participants were encouraged to share current DS consumption with the interviewers to include product label information, dose, strength, and frequency.

Across two consecutive NHANES cycles (2009–2010, 2011–2012), we combined dietary and individual DS intakes reported over two 24-hour periods [17] with the food patterns equivalent database from the U.S. Department of Agriculture (USDA) as well as the ILSI North America Fortification database (see: http://ilsina.org/our-work/nutrition/fortification/). Since this study was a secondary data analysis of publicly available federal data, Human Subject Institutional Review Board approval was not required by the Medical University of South Carolina.

Twenty-four hour dietary intake data were complete and available for 20,293 participants. The sample for analysis included a total of 16,975 participants two years of age and older (see Table S1). Individuals with incomplete data (*n* = 1947) and those under two years of age (*n* = 1371) were excluded.

Food and beverage consumption reported in the 2009–2010 and 2011–2012 NHANES dietary interview component (What We Eat in America, WWEIA) and nutrient estimates were obtained using the USDA’s Food and Nutrient Database for Dietary Studies (FNDDS) [18]. Estimates for nutrients as naturally occurring (i.e., intrinsic or all foods and beverages excluding E/F), enriched, and fortified were available through the ILSI North America Fortification database. The ILSI North America Fortification database was combined with the two 24-hour individual food intakes from NHANES, demographics data, and total Supplement Files. 

The number of grams consumed per participant from the NHANES food file was multiplied by the nutrient proportion in the ILSI North America Fortification database, and divided by 100 to determine the amount of consumed grams of study nutrients for each level (naturally occurring, E/F added, DSs added) in the specified food/beverage. The total nutrient intake (per 100 g) for each nutrient as naturally occurring, enriched/fortified, and DSs was then calculated by summing each of the above levels for every participant from the various foods and beverages consumed. 

### 2.2. Estimation of Usual Intake, %<EAR, and % ≥UL

The NCI usual intake estimation methodology was utilized to estimate the prevalence of usual intake based on data from two 24-hour dietary recalls [14,15]. Estimation of usual intake at the individual level is possible through the NCI’s MIXTRAN and DISTRIB computer macro. The usual intake macro was run to estimate total nutrient intake following the addition of DSs’ nutrient intake. Analyses controlled for age, interview day (1st vs. 2nd), PIR (<131%, 131–185%, >185%), weekend day (yes/no), and race/ethnicity (NHW, NHB, Hispanic, and other races/ethnicities).

Three cumulative sources were used to generate estimated usual intake data for the 15 nutrients of interest, which were those included in the ILSI North America Fortification database: a) foods and beverages as naturally occurring, b) foods and beverages including E/F, and c) foods and beverages including E/F and DSs. These nutrients included vitamins A, D, E, C, B_6_, and B_12_, folate, thiamin, riboflavin, niacin, iron, zinc, calcium, magnesium, and potassium. For folic acid and folate, dietary folate equivalents (DFE) was used [19]. Percent below the EAR (%>AI for potassium), and % ≥UL were estimated as appropriate. We estimated the proportion of the population with nutrient intakes from sources as naturally occurring, enriched/fortified, and supplements greater than or equal to the ULs with the exception of thiamin, riboflavin, potassium, and vitamins A, E, and B_12_, for which ULs have not been estimated [19,20,21]. The ULs for niacin and magnesium were derived from non-food sources (enriched/fortified or DSs) [19,22]. 

### 2.3. Statistical Analysis

We accounted for the NHANES clustered sampling design and oversampling in all analyses and adjusted for differential non-coverage and non-response across the two continuous NHANES cycles [23,24,25]. Frequencies were reported for sample size from day 1 and day 2 interviews. Means and standard errors (SE) were calculated for average usual intake, average %<EAR (%>AI for potassium), and average %≥UL. SEs were estimated using Balanced Repeated Replication and NHANES weights were applied. The age group analyzed was ≥2 years for both sexes combined. PIR was defined by the following categories: <131%, 131% to 185%, and >185% of the poverty index, which is similar to PIR cut-points used by the DGAC 2015–2020, SNAP eligibility of ≤130% of the poverty line, and past studies [7,26]. Linear mixed models were used to evaluate race/ethnic differences by PIR category and within each level of nutrient intake. NHW was used as the race/ethnic reference category.

For ease of interpretation, bar graphs of the percent below EAR are presented for all nutrients stratified by race/ethnicity and PIR category. All analyses were conducted using SAS, version 9.4, and its complex survey-specific procedures (SAS Institute; Cary, NC, USA) and a *p*-value < 0.05 was considered statistically significant.

## 3. Results

### 3.1. Estimated Mean Usual Nutrient Intakes from Foods and Beverages as Naturally Occurring, Plus Enriched/Fortified, and Plus Dietary Supplement Sources

Estimated mean usual nutrient intakes and %<EAR are displayed in Table 1 for nutrients by three levels (as naturally occurring, with E/F, and with DSs) and by racial/ethnic group. Usual nutrient intake appeared to differ by race/ethnicity over the three levels of nutrient intake. Across all races/ethnicities and levels, %<EAR was highest overall for vitamins A, D, E, and C as well as calcium and magnesium. NHBs appeared to have the highest %<EAR for 13 of the 14 nutrients, and the lowest %>AI for potassium compared with the three other racial/ethnic groups. For vitamin C, the %<EAR was similar (27%) for NHWs and NHBs. A decrease was observed across racial/ethnic groups for the %<EAR for vitamin D from 100% as naturally occurring for all groups to 91%–97% after E/F. However, after the addition of DSs, the %<EAR for vitamin D decreased further for all groups (93% to 65% for NHWs, 95% to 80% for NHBs, 91% to 79% for Hispanics, and 94% to 74% for the other race/ethnicity group). An appreciable decline in the %<EAR was also observed for folate for all groups following E/F that further decreased after considering DSs. Specifically, the %<EAR for folate from naturally occurring, E/F, and DSs sources was 82%, 7%, and 5% for NHWs; 89%, 14%, and 12% for NHBs; 78%, 6%; and 5% for Hispanics; and 78%, 5%, and 4% for the other race/ethnicity group, respectively. Within racial/ethnic groups vitamin A also exhibited large declines in the %<EAR when E/F sources were included, declining from 65% to 32% for NHWs; 76% to 50% for NHBs; 66% to 39% for Hispanics; and 63% to 37% for the other race/ethnicity group. Iron intake for NHWs was higher and %<EAR was lower than the other three races/ethnicities. 

### 3.2. Total Usual Mean Nutrient Intakes by Race/Ethnicity and PIR Category

Total usual mean nutrient intakes by intake level are presented by racial/ethnic group within PIR category (see Table 2). Focusing on the usual intake of nutrients as naturally occurring, comparing intake of folate within PIR categories across race/ethnic groups, usual intake of folate was lowest for NHBs in each PIR category. For example, in the lowest PIR category, mean (SE) usual intake of folate (ug/DFE) was 159 (0.2) in NHBs compared to 193 (0.2) in NHWs, 192 (0.2) in Hispanics, and 192 (0.2) in the other race/ethnic group. Moreover, intake of folate was highest for NHWs in the lowest PIR category and highest for Hispanics in the middle and highest PIR categories. Similarly, calcium intake was lowest for NHBs compared to NHWs, Hispanics, and the other race/ethnic group, across all PIR categories, but highest for NHWs compared to NHBs, Hispanics, and the other race/ethnic group across all PIR categories.

Intake of folate with E/F was lowest for NHBs comparing intake of folate within PIR categories across all race/ethnic groups for each PIR category. For example, in the lowest PIR category, mean (SE) usual intake of folate with E/F (ug/DFE) was 458 (0.4) in NHBs compared to 533 (0.4) in NHWs, 536 (0.4) in Hispanics, and 550 (0.8) in the other race/ethnic group. Intake of folate with E/F was highest for the other race/ethnic group, comparing intake of folate within PIR categories across all race/ethnic groups for each PIR category. This pattern was also seen for the usual nutrient intake of calcium with E/F intake lowest for NHBs across all PIR categories and highest for NHWs across all PIR categories. 

With the addition of nutrients from DSs, the usual intakes of folate was lowest for NHBs in all PIR categories and highest for NHWs in all PIR categories. Calcium followed a similar pattern, with the usual intake of calcium including DSs lowest for NHBs in all PIR categories and highest for NHWs in all PIR categories.

### 3.3. Percent Less than the EAR for Nutrients by Race/Ethnicity and PIR Category

**Figure 1:**
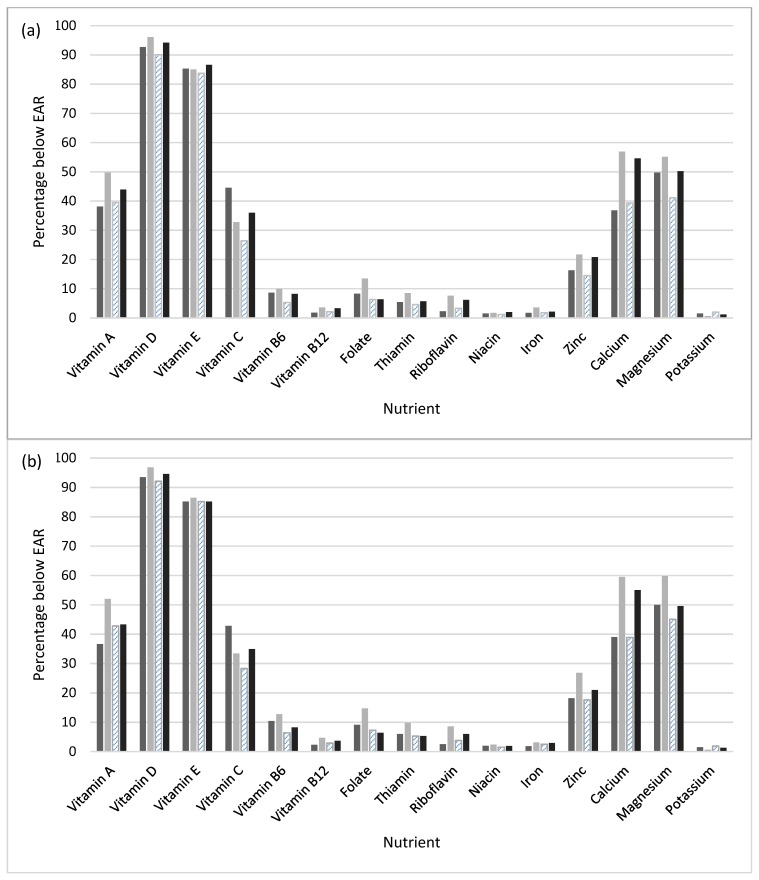

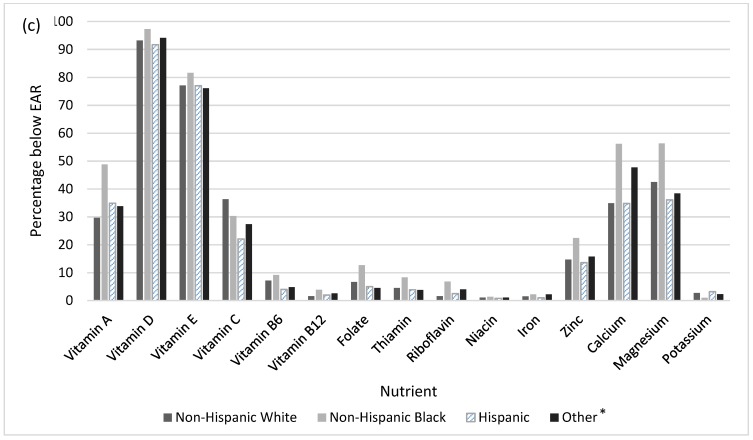
Percent less than the estimated average requirement (EAR) for naturally occurring plus enriched/fortified nutrient intake by race/ethnicity and poverty index ratio (PIR) category: (**a**) <131%, (**b**) 131–85%, and (**c**) >185%. * The other race/ethnicity group included non-Hispanic persons reporting multiple races.

The %<EAR for the three nutrient levels is shown based on race/ethnicity and PIR category (see Table 3 and Figure 1a–c). The %<EAR for folate as naturally occurring was highest for NHBs compared to NHWs, Hispanics, and the other race/ethnic group in all PIR categories, and lowest for Hispanics compared to NHWs, NHBs, and the other race/ethnic group in all PIR categories. For example, in the lowest PIR category, the average %<EAR (SE) for folate as naturally occurring was 89.8% (0.6) in NHBs compared to 86.5% (1.0) in NHWs, 79.7% (1.6) in Hispanics, and 83.5% (1.8) in the other race/ethnic group. Compared with NHWs, the %<EAR for folate as naturally occurring was significantly higher for NHBs and significantly lower for Hispanics regardless of PIR category (*p* < 0.0001 for all). The %<EAR for calcium as naturally occurring was highest for NHBs in all PIR categories, and lowest for NHWs in all PIR categories. The %<EAR for calcium as naturally occurring for NHBs was significantly higher than NHWs in all PIR categories (*p* < 0.0001 for all). 

When E/F of foods is added, the %<EAR for folate with E/F was highest for NHBs in all PIR categories. The %<EAR for folate with E/F was lowest for Hispanics in the lowest PIR category, and for the other race/ethnic group in the middle and highest PIR categories. For example, in the lowest PIR category, the average %<EAR (SE) for folate with E/F was 13.5% (1.4) in NHBs compared to 8.3% (1.0) in NHWs, 6.3% (0.8) in Hispanics, and 6.4% (1.4) in the other race/ethnic group. The %<EAR for folate as naturally occurring was significantly higher for NHBs than NHW counterparts, and significantly lower for Hispanics in the lowest PIR category, and the other race/ethnic group in the middle and highest PIR categories (*p* < 0.0001 for all). The %<EAR for calcium with E/F was highest for NHBs in all PIR categories, and lowest for NHWs in the lowest PIR category and for Hispanics in the middle and highest PIR categories. The %<EAR for calcium with E/F was significantly higher for NHBs in all PIR categories than NHWs (*p* < 0.0001).

With the addition of estimated nutrient intake from DS use, the %<EAR for folate with DSs was highest for NHBs in all PIR categories and lowest for the other race/ethnicity group in all PIR categories. Compared with NHWs in all PIR categories, the %<EAR for folate with DSs was significantly higher for NHBs and significantly lower for the other race/ethnicity group (*p* < 0.0001 for all). The %<EAR for calcium with DSs was highest for NHBs in all PIR categories and lowest for NHWs in all PIR categories. The %<EAR for calcium plus DSs for NHBs was significantly higher than NHWs in all PIR categories (*p* < 0.0001 for all).

### 3.4. Percent Greater than or Equal to the UL for Nutrients by Race/Ethnicity and PIR Category

Table 4 shows the % ≥UL for the four racial/ethnic groups by PIR category and nutrient level for nutrients for which the UL is available. Focusing on usual intake of nutrients as naturally occurring, the % ≥UL was ≤2% for vitamin A, folate, and calcium. For usual intake of nutrients with E/F, the % ≥UL was ≤10.1% for vitamin A, niacin, zinc, and calcium. Following the addition of DSs, the % ≥UL was ≤10% for vitamins D, C, B_6_, calcium, and magnesium. When considering usual intake with DSs, the % ≥UL for folate ranged from 24.4% to 42.6% and the % ≥UL for zinc ranged from 5.4% to 12% across race/ethnic groups and PIR categories.

## 4. Discussion

In summary, usual intake levels of vitamins A, C, B_6_, folate, thiamin, riboflavin, iron, zinc, calcium, magnesium, and potassium were lowest in NHBs when compared to NHWs, Hispanics, and the other race/ethnic group, even after accounting for PIR category. Usual intake levels of vitamins A, E, B_12_, riboflavin, niacin, calcium, and potassium were consistently highest in NHWs when compared to NHBs, Hispanics, and the other race/ethnicity group across all PIR categories. The usual nutrient intake levels below the EAR also differed across racial/ethnic groups and within PIR categories. Compared with NHWs, Hispanics, and the other race/ethnic group, the %<EAR was highest in NHBs for vitamins A, B_12_, folate, thiamin, riboflavin, zinc, calcium, and magnesium, whereas the %<EAR was lowest in NHBs for potassium, across all PIR categories. These results are of particular concern because other studies have demonstrated the highly continued prevalence of neural tube defects among infants of young women from inner city low income NHB and U.S. southern state Hispanic populations [29,30]. The data indicate a lack of knowledge about the increased need for folic acid during pregnancy. Based on our data and these reports, targeting young, lower economic women of childbearing age from these populations with educational information to encourage them to consume adequate synthetic folic acid daily (from fortified foods or supplements) in addition to food forms of folate from a varied diet is needed.

In NHWs, the %<EAR was lowest for vitamin B_12_ and riboflavin compared to NHBs, Hispanics, and the other race/ethnic group. The %<EAR was highest for magnesium and lowest for vitamins C, B_6_, and magnesium in Hispanics compared to NHWs, NHBs, and the other race/ethnic group. With regard to the UL, the usual intake exceeded UL by ≥10% for folate and zinc with E/F. The % ≥UL for folate ranged from 10.5% to 27.1% and the % ≥UL for zinc ranged from 3.8% to 10.1% across racial/ethnic groups and PIR categories. The addition of use of DSs further increased the % ≥UL for both nutrients and especially folate for which the % ≥UL ranged from 24.4% to 42.6%. 

Nutrient intakes of certain vitamins and minerals in the U.S. population have increased as a result of fortification and enrichment of foods in the U.S. food supply even though only a few studies to date have focused on E/F [5,11,12] and studies that also focused on race/ethnicity [9,10] or PIR categories [7,8,9] have been limited in scope. In 2011, Fulgoni et al. utilized the ILSI North America Fortification database to assess E/F for the population two years of age and older, as well as children and adults by age group using NHANES 2003–2006 [5]. Similar to findings from our study involving the 2009–2012 NHANES, Fulgoni et al. reported that DRI-recommended nutrient intake levels were not met by many Americans prior to E/F and DS use [5]. Specifically, intakes of vitamins A, C, and D, thiamin, iron, and folate greatly improved with E/F. We examined nutrient intake levels by race/ethnicity within PIR category and observed increased intakes of the same nutrients with E/F. Fulgoni et al. did not report on racial/ethnic differences in their earlier study.

DS use and nutrient intakes and inadequacies were also evaluated by race/ethnicity among adults 19 years of age and older by Blumberg et al. using the NHANES 2009–2012. However, dietary information for NHA was limited to the NHANES 2011–2012 cycle [10]. Compared with NHBs, Hispanics, and NHAs, NHWs were found to have half as many nutrient inadequacies [10]. Within each racial/ethnic group (NHW, NHB, Hispanic [Mexican Americans and other Hispanics], and NHA), intakes of most of the 19 nutrients were higher for DS users compared to non-users [10]. A second study that used NHANES 2009–2012 data examined vegetable consumption in women of childbearing age [9]. While comprehensive, these studies did not examine the impact of E/F. 

While dietary guidelines have been in place for years, adherence to many food groups has been low and racial/ethnic differences have been observed. Among African-Americans, Latinos born in the U.S., and Latinos born in Mexico or South America who participated in the Multiethnic Cohort Study in Hawaii and Los Angeles, the group with the lowest adherence to the Food Guide Pyramid recommendations were African-Americans followed by U.S.-born Latinos [31]. Among the three racial/ethnic groups, adherence to the Food Guide Pyramid recommendations for dairy was lowest among all the food groups. Recently, food groups consumed in the diets of Mexicans, Mexican Americans born in the U.S. or Mexico, and NHWs were assessed for food acculturation using one 24-hour dietary recall from the Mexican National Nutrition Survey 1999 and NHANES 1999–2006 [32]. Included in the study were female adolescents and adults 12–49 years of age, as well as children 2–11 years of age. Specifically, desserts, salty snacks, pizza, French fries, low-fat meat and fish, high-fiber bread, low-fat milk, saturated fat, and sugar intakes were higher for the three U.S. groups than for Mexicans [32]. Intakes of corn tortillas, low-fiber bread, high-fat milk, and Mexican fast food were lower for the three U.S. groups. When examining food group and nutrient intakes among the three U.S. groups and by age, similar patterns were observed [32]. In terms of the timing of acculturation, the findings indicated that “within one generation in the U.S.” the positive aspects of the purely Mexican diet were replaced by selections of higher fat and energy U.S. diet components [32]. These acculturation data, coupled with what was reported here, suggest that nutrition education targeted to new immigrant groups in the U.S., particularly children, might have strong benefits for reducing a potential lifelong impact of U.S.-acquired poor dietary patterns.

Overconsumption of energy from added sugars and solid fats was observed for individuals two years of age and older across all racial/ethnic (NHW, NHB, Mexican American) and income (lowest, middle, highest) groups using the NHANES 2001–2004 in a 2012 study by Kirkpatrick et al. [26]. The authors reported increased adherence to most food groups according to dietary guideline recommendations for those with the highest incomes (>185% of poverty threshold), of whom adults were twice as likely to consume the minimum recommendations for milk, oils, and total vegetables than those with the lowest incomes (≤130%) [26]. NHBs were the least likely to meet dietary guideline recommendations, and when limited to children, the same was true for the recommended minimum milk consumption (15% compared with 42% and 35% for NHWs and Mexican-American children, respectively) [26]. Among adults, only 2% of both NHWs and NHBs consumed the recommended minimum of dry beans and peas compared with 20% of Mexican-Americans [26].

Similar to past findings [33], compared to the lowest and middle income groups, adherence to dietary guideline recommendations for several food groups (e.g., whole grains, total vegetables, and milk) was observed to be higher for adults with the highest income [26]. Barriers and facilitators or promoters for consumption of certain food groups including fruits and vegetables have been previously described for diverse populations. Among African-Americans, barriers included cost and finances (for all foods) and preferences (for fruits and fast food) [34], as well as access to fresh produce [35], whereas facilitators/promoters included taste and health concerns [34], with differences reported by age and gender. In focus groups involving Hispanics, African-Americans, and Caucasians, lack of time especially among those less than 50-years-old, cost, rates of food spoiling, media’s fast-food promotion, and convenience of pre-packaged foods were reported as barriers to fruit and vegetable intake, while knowledge of health benefits and children’s health were facilitators [35]. A barrier for Hispanics was the impact of the U.S. culture on food availability [35]. 

Limitations of our study include use of dietary information from two 24-hour NHANES recalls to estimate nutrient intake, which may be subject to bias (recall and interviewer), although usual intake was assessed given the use of two 24-hour recalls. Past studies involving NHANES 24-hour recall data have been criticized as having underestimation of the overall intake and nutrient levels by U.S. adults due to food and drink item portion size underreporting [36]. The current study was able to better estimate the sources of nutrients consumed by the U.S. population through the incorporation of the ILSI North America Fortification database that contains naturally occurring ( intrinsic), enriched, and fortified nutrient estimates for foods and beverages coded using the USDA’s FNDDS based on reported consumption in the 2009–2010 and 2011–2012 NHANES. There are many strengths of the current study including use of nationally-representative dietary databases (NHANES and FNDDS) that contain the most comprehensive dietary intake information available for the U.S. population, as well as use of the USDA’s AMPM approach for collection of dietary intake data by NHANES to limit misreporting of food and beverage items [18,37]. The selected PIR categories were similar to the DGAC 2015–2020 PIR cut points, eligibility for SNAP of ≤130% the poverty line, and that of past studies [7,26].

## 5. Conclusions

Fortification and enrichment of food, as well as the use of DSs play an important role in meeting nutrient intake recommendations and reducing inadequacies. However, nutrient intakes have been shown to vary by demographic characteristics including race/ethnicity and income. Even after foods were enriched/fortified and DSs were added to the diet, over half of the population failed to meet the EAR for vitamins D (59.7% to 84.4%) and E (76.1% to 86.6%) with ~50% of NHBs not meeting the EAR for vitamin A, calcium, and magnesium. Disparities in access to foods containing nutrients found in enriched/fortified and DS sources impact nutrient intakes and intakes below the EAR can result in negative health outcomes, particularly for the under-consumed shortfall nutrients reported in the 2015 DGAC report. Future studies can further investigate racial/ethnic differences in DRIs by age group and specific nutrient sources. Our study further underscored intake insufficiencies of the same shortfall nutrients identified by the last DGAC. Going forward, educational resources and programs geared toward increased intake of these nutrients by population groups at highest risk for deficiency such as children, women of child-bearing age, and the elderly should continue to be emphasized to improve dietary patterns, while addressing barriers including the convenience of fast-food and promotion by the media.

## Figures and Tables

**Table 1:** Usual nutrient intake and percent less than the estimated average requirement for the U.S. population 2 years of age and older by race/ethnicity, NHANES 2009–2012 ^1–3^.

Race/Ethnicity								
	Non-Hispanic White		Non-Hispanic Black		Hispanic		Other Race/Ethnicity ^4^	
Nutrient	Usual Intake	%<EAR	Usual Intake	%<EAR	Usual Intake	%<EAR	Usual Intake	%<EAR
**Vitamin A**, µg RAE/day ^5–6^								
Naturally occurring	456 ± 0.2	64.8 ± 2.0	366 ± 0.2	75.6 ± 1.8	406 ± 0.2	66.2 ± 1.4	435 ± 0.4	62.7 ± 2.0
+ Enriched/fortified	689 ± 0.4	31.8 ± 1.8	529 ± 0.4	49.9 ± 2.4	591 ± 0.4	39.2 ± 1.4	620 ± 0.6	37 ± 1.8
+ Dietary supplements	---	---	---	---	---	---	---	---
**Vitamin D**, µg/day ^7–8^								
Naturally occurring	1.6 ± 0.0	100 ± 0.0	1.7 ± 0.0	100 ± 0.0	1.7 ± 0.0	100 ± 0.0	1.6 ± 0.0	100 ± 0.0
+ Enriched/fortified	5.4 ± 0.0	93.0 ± 0.8	4.5 ± 0.0	96.6 ± 0.4	5.7 ± 0.0	91.2 ± 0.6	5.1 ± 0.0	94.2 ± 1.2
+ Dietary supplements	14.2 ± 0.0	64.5 ± 0.6	9.5 ± 0.0	80.1 ± 0.4	8.3 ± 0.0	79.0 ± 0.6	10.5 ± 0.0	71.4 ± 1.0
**Vitamin E**, mg AT/day ^6,9^								
Naturally occurring	7.5 ± 0.0	85.9 ± 0.6	6.8 ± 0.0	88.2 ± 0.6	6.6 ± 0.0	87.1 ± 1.2	6.9 ± 0.0	86.2 ± 1.4
+ Enriched/fortified	8.3 ± 0.0	79.3 ± 0.8	7.2 ± 0.0	84.2 ± 0.6	7.1 ± 0.0	82.6 ± 1.2	7.5 ± 0.0	80.5 ± 1.8
+ Dietary supplements	---	---	---	---	---	---	---	---
**Vitamin C**, mg/day ^9^								
Naturally occurring	62.9 ± 0.0	53.1 ± 1.4	55.1 ± 0.0	56.2 ± 1.8	67.1 ± 0.0	41.8 ± 1.4	67.3 ± 0.0	43.4 ± 2.8
+ Enriched/fortified	82.1 ± 0.0	38.1 ± 1.0	86.1 ± 0.0	32.0 ± 1.8	94.3 ± 0.0	25.3 ± 1.4	89.1 ± 0.2	29.6 ± 2.2
+ Dietary supplements	156 ± 0.4	27.3 ± 0.6	125 ± 0.6	27.0 ± 1.4	121 ± 0.2	22.1 ± 1.2	142 ± 0.4	23.0 ± 1.6
**Vitamin B_6_**, mg/day ^10^								
Naturally occurring	1.6 ± 0.0	16.9 ± 0.8	1.5 ± 0.0	18.1 ± 1.0	1.6 ± 0.0	11.1 ± 1.2	1.6 ± 0.0	11.0 ± 1.0
+ Enriched/fortified	2.0 ± 0.0	7.4 ± 0.6	1.8 ± 0.0	9.9 ± 0.8	2.0 ± 0.0	5.2 ± 0.8	1.9 ± 0.0	6.0 ± 0.6
+ Dietary supplements	4.9 ± 0.0	4.8 ± 0.4	3.0 ± 0.0	8.0 ± 0.6	3.1 ± 0.0	4.4 ± 0.6	3.5 ± 0.0	4.5 ± 0.6
**Vitamin B_12_**, µg/day ^10^								
Naturally occurring	4.2 ± 0.0	4.0 ± 0.4	3.7 ± 0.0	7.0 ± 0.8	3.9 ± 0.0	4.9 ± 0.6	3.8 ± 0.0	5.4 ± 0.8
+ Enriched/fortified	5.5 ± 0.0	1.7 ± 0.2	4.6 ± 0.0	3.9 ± 0.6	5.0 ± 0.0	2.4 ± 0.4	4.8 ± 0.0	3.0 ± 0.4
+ Dietary supplements	44.6 ± 0.4	1.1 ± 0.2	21.2 ± 0.2	3.2 ± 0.4	17.9 ± 0.2	2.1 ± 0.4	29.1 ± 0.4	2.3 ± 0.2
**Folate**, µg DFE/day ^10,11^								
Naturally occurring	216 ± 0.2	82.2 ± 0.8	177 ± 0.2	89.2 ± 0.6	207 ± 0.2	78.3 ± 1.4	212 ± 0.2	77.9 ± 1.8
+ Enriched/fortified	559 ± 0.2	7.3 ± 0.6	475 ± 0.2	13.8 ± 1.0	551 ± 0.2	6.3 ± 0.8	576 ± 0.4	5.3 ± 1.0
+ Dietary supplements	765 ± 0.6	5.1 ± 0.4	588 ± 0.6	11.5 ± 0.8	642 ± 0.6	5.4 ± 0.6	725 ± 1.0	4.0 ± 0.8
**Thiamin**, mg/day ^10^								
Naturally occurring	1.0 ± 0.0	40.4 ± 1.6	0.8 ± 0.0	55.0 ± 1.4	0.9 ± 0.0	41.4 ± 1.6	0.9 ± 0.0	39.9 ± 2.8
+ Enriched/fortified	1.7 ± 0.0	4.8 ± 0.4	1.5 ± 0.0	8.7 ± 0.6	1.6 ± 0.0	4.7 ± 0.6	1.6 ± 0.0	4.5 ± 0.6
+ Dietary supplements	4.8 ± 0.0	3.4 ± 0.2	2.4 ± 0.0	7.3 ± 0.6	2.5 ± 0.0	4.1 ± 0.4	3.3 ± 0.0	3.6 ± 0.6
**Riboflavin**, mg/day ^10^								
Naturally occurring	1.6 ± 0.0	10.0 ± 0.6	1.2 ± 0.0	26.6 ± 1.2	1.4 ± 0.0	14.5 ± 1.2	1.4 ± 0.0	16.5 ± 1.6
+ Enriched/fortified	2.2 ± 0.0	1.8 ± 0.2	1.7 ± 0.0	7.5 ± 0.6	2.0 ± 0.0	3.3 ± 0.4	1.9 ± 0.0	4.9 ± 0.6
+ Dietary supplements	4.1 ± 0.0	1.4 ± 0.2	2.5 ± 0.0	6.5 ± 0.6	2.8 ± 0.0	2.9 ± 0.4	3.0 ± 0.0	4.0 ± 0.4
**Niacin**, mg/day ^10^								
Naturally occurring	17.7 ± 0.0	8.7 ± 0.6	16.6 ± 0.0	9.9 ± 0.8	16.3 ± 0.0	9.5 ± 1.0	16.5 ± 0.0	9.5 ± 1.2
+ Enriched/fortified	24.8 ± 0.0	1.2 ± 0.2	22.9 ± 0.0	1.7 ± 0.2	23.4 ± 0.0	1.2 ± 0.2	23.1 ± 0.0	1.4 ± 0.2
+ Dietary supplements	34.1 ± 0.0	0.8 ± 0.2	27.1 ± 0.0	1.4 ± 0.2	26.2 ± 0.0	1.1 ± 0.2	27.8 ± 0.0	1.1 ± 0.2
**Iron**, mg/day ^5^								
Naturally occurring	17.7 ± 0.0	8.7 ± 0.6	8.3 ± 0.0	19.6 ± 1.0	8.9 ± 0.0	13.9 ± 1.0	8.9 ± 0.0	15.7 ± 1.8
+ Enriched/fortified	24.8 ± 0.0	1.2 ± 0.2	13.7 ± 0.0	3.0 ± 0.2	15.1 ± 0.0	1.7 ± 0.2	14.6 ± 0.0	2.3 ± 0.2
+ Dietary supplements	34.1 ± 0.0	0.8 ± 0.2	16.2 ± 0.0	2.5 ± 0.2	16.9 ± 0.0	1.3 ± 0.2	16.8 ± 0.0	1.6 ± 0.2
**Zinc**, mg/day ^5^								
Naturally occurring	10.4 ± 0.0	21.0 ± 1.2	9.1 ± 0.0	28.9 ± 1.4	9.9 ± 0.0	19.3 ± 1.2	9.6 ± 0.0	22.8 ± 1.8
+ Enriched/fortified	11.5 ± 0.0	15.2 ± 1.0	9.9 ± 0.0	23.3 ± 1.4	10.9 ± 0.0	15.2 ± 0.8	10.6 ± 0.0	18.1 ± 1.4
+ Dietary supplements	15.3 ± 0.0	11.0 ± 0.8	11.8 ± 0.0	20.0 ± 1.2	12.4 ± 0.0	13.5 ± 0.8	13.0 ± 0.0	14.6 ± 1.2
**Calcium**, mg/day ^7–8^								
Naturally occurring	984 ± 0.4	42.8 ± 1.0	778 ± 0.4	65.4 ± 1.6	943 ± 0.4	46.1 ± 1.6	828 ± 0.6	58.1 ± 2.4
+ Enriched/fortified	1065 ± 0.4	35.6 ± 0.8	850 ± 0.6	57.3 ± 1.6	1025 ± 0.6	38.3 ± 1.4	897 ± 0.8	50.6 ± 2.6
+ Dietary supplements	1213 ± 0.6	26.5 ± 0.6	914 ± 0.6	51.5 ± 1.6	1083 ± 0.6	34.3 ± 1.2	981 ± 1.0	43.4 ± 2.4
**Magnesium**, mg/day ^8^								
Naturally occurring	292 ± 0.2	45.8 ± 1.2	243 ± 0.2	57.2 ± 1.2	278 ± 0.2	41.0 ± 1.4	277 ± 0.2	43.8 ± 1.8
+ Enriched/fortified	296 ± 0.2	44.5 ± 1.2	245 ± 0.2	56.5 ± 1.2	281 ± 0.2	40.3 ± 1.4	280 ± 0.2	43.0 ± 2.0
+ Dietary supplements	320 ± 0.2	38.5 ± 1.2	256 ± 0.2	53.2 ± 1.2	289 ± 0.2	38.0 ± 1.4	291 ± 0.2	39.7 ± 2.0
**Potassium**, mg/day ^12,13^		**% >AI**		**% >AI**		**% >AI**		**% >AI**
Naturally occurring	2668 ± 1.0	2.2 ± 0.4	2239 ± 1.2	0.7 ± 0.0	2539 ± 1.2	2.2 ± 0.2	2477 ± 1.8	1.7 ± 0.4
+ Enriched/fortified	2689 ± 1.0	2.3 ± 0.4	2253 ± 1.2	0.8 ± 0.0	2550 ± 1.2	2.3 ± 0.2	2492 ± 1.8	1.8 ± 0.4
+ Dietary supplements	2703 ± 1.0	2.4 ± 0.4	2259 ± 1.2	0.8 ± 0.0	2555 ± 1.2	2.3 ± 0.2	2498 ± 1.8	1.8 ± 0.4

^1^ Mean ± standard error. ^2^ Data Source: What We Eat in America, NHANES 2009–2012 [17]. ^3^ Usual intake distribution estimated using the National Cancer Institute Method for individuals 2 years of age and older, including pregnant and lactating women. Accessible via https://epi.grants.cancer.gov/diet/usualintakes/method.html. ^4^ Other race/ethnicity included non-Hispanic persons reporting multiple races. ^5^ Dietary Reference Intakes for Vitamin A, Vitamin K, Arsenic, Boron, Chromium, Copper, Iodine, Iron, Manganese, Molybdenum, Nickel, Silicon, Vanadium, and Zinc (2001) [21]. ^6^ A supplements file is not currently available for Vitamins A and E in NHANES for 2009–2012, and it will be released at a later date. ^7^ Dietary Reference Intakes for Calcium and Vitamin D (2011) [27]. ^8^ Dietary Reference Intakes for Calcium, Phosphorous, Magnesium, Vitamin D, and Fluoride (1997) [22]. ^9^ Dietary Reference Intakes for Vitamin C, Vitamin E, Selenium, and Carotenoids (2000) [28]. ^10^ Dietary Reference Intakes for Thiamin, Riboflavin, Niacin, Vitamin B_6_, Folate, Vitamin B_12_, Pantothenic Acid, Biotin, and Choline (1998) [19]. ^11^ Folate EAR is presented as dietary folate equivalents (DFE). 1 DFE = 1 µg food folate = 0.6 µg of folic acid from fortified food or supplement consumed with food = 5 µg of a supplement taken on an empty stomach. ^12^ Dietary Reference Intakes for Water, Potassium, Sodium, Chloride, and Sulfate (2005) [20]. ^13^ The AI approach was used for potassium. U.S., United States. NHANES, National Health and Nutrition Examination Survey. EAR, estimated average requirement. RAE, retinol activity equivalents. AT, a-tocopherol. DFE, dietary folate equivalents. AI, adequate intake.

**Table 2:** Usual nutrient intake for the U.S. population 2 years of age and older by race/ethnicity and poverty index ratio category, NHANES 2009–2012 ^1–3^.

	Non-Hispanic White			Non-Hispanic Black			Hispanic			Other Race/Ethnicity ^4^		
	PIR Category			PIR Category			PIR Category			PIR Category		
Nutrient	<131%	131–185%	>185%	<131%	131–185%	>185%	<131%	131–185%	>185%	<131%	131–185%	>185%
**Vitamin A**, µg RAE/day ^5,6^												
Naturally occurring	406 (0.4)	422 (0.6)	476 (0.4)	336 (0.4)	354 (0.6)	402 (0.4)	383 (0.4)	400 (0.6)	453 (0.4)	393 (0.8)	404 (1.2)	455 (0.6)
+ Enriched/fortified	624 (0.6)	646 (1.0)	714 (0.4)	498 (0.6)	517 (1.0)	568 (0.6)	569 (0.6)	580 (1.0)	647 (0.6)	567 (1.0)	578 (1.6)	643 (0.8)
+ Dietary supplements	--	--	--	--	--	--	--	--	--	--	--	--
**Vitamin D**, µg/day ^7,8^												
Naturally occurring	1.5 (0.0)	1.5 (0.0)	1.7 (0.0)	1.5 (0.0)	1.6 (0.0)	1.8 (0.0)	1.6 (0.0)	1.7 (0.0)	1.9 (0.0)	1.5 (0.0)	1.5 (0.0)	1.6 (0.0)
+ Enriched/fortified	5.5 (0.0)	5.3 (0.0)	5.4 (0.0)	4.6 (0.0)	4.4 (0.0)	4.3 (0.0)	5.9 (0.0)	5.6 (0.0)	5.7 (0.0)	5.1 (0.0)	5.0 (0.0)	5.2 (0.0)
+ Dietary supplements	11.6 (0.2)	11.6 (0.0)	14.7 (0.0)	9.0 (0.2)	7.7 (0.0)	10.9 (0.0)	7.9 (0.0)	7.3 (0.0)	9.5 (0.0)	8.1 (0.0)	9.8 (0.0)	12.4 (0.0)
**Vitamin E**, mg AT/day ^6,9^												
Naturally occurring	6.6 (0.0)	6.9 (0.0)	7.9 (0.0)	6.1 (0.0)	6.5 (0.0)	7.6 (0.0)	6.2 (0.0)	6.6 (0.0)	7.5 (0.0)	6.1 (0.0)	6.4 (0.0)	7.3 (0.0)
+ Enriched/fortified	7.2 (0.0)	7.5 (0.0)	8.7 (0.0)	6.5 (0.0)	6.9 (0.0)	8.2 (0.0)	6.6 (0.0)	7.0 (0.0)	8.2 (0.0)	6.7 (0.0)	6.9 (0.0)	8.0 (0.0)
+ Dietary supplements	--	--	--	--	--	--	--	--	--	--	--	--
**Vitamin C**, mg/day ^9^												
Naturally occurring	50.6 (0.0)	56.8 (0.2)	67.1 (0.0)	47.1 (0.0)	53.9 (0.2)	64.5 (0.2)	59.5 (0.0)	66.1 (0.2)	78.8 (0.2)	56.8 (0.2)	60.8 (0.2)	71.6 (0.2)
+ Enriched/fortified	71.4 (0.0)	75.8 (0.2)	85.6 (0.0)	79.4 (0.2)	84.0 (0.2)	93.8 (0.2)	88.7 (0.2)	91.9 (0.2)	104.6 (0.2)	79.4 (0.2)	81.7 (0.4)	92.8 (0.2)
+ Dietary supplements	116 (0.4)	137 (0.8)	177 (0.6)	99.2 (0.4)	107 (0.6)	161 (1.4)	103 (0.2)	114 (0.4)	152 (0.6)	109 (0.6)	157 (2.6)	156 (0.6)
**Vitamin B_6_**, mg/day ^10^												
Naturally occurring	1.5 (0.0)	1.5 (0.0)	1.6 (0.0)	1.4 (0.0)	1.4 (0.0)	1.6 (0.0)	1.5 (0.0)	1.6 (0.0)	1.7 (0.0)	1.5 (0.0)	1.5 (0.0)	1.7 (0.0)
+ Enriched/fortified	1.9 (0.0)	1.9 (0.0)	2.1 (0.0)	1.7 (0.0)	1.7 (0.0)	1.9 (0.0)	1.9 (0.0)	1.9 (0.0)	2.2 (0.0)	1.8 (0.0)	1.8 (0.0)	2.0 (0.0)
+ Dietary supplements	3.5 (0.0)	4.1 (0.0)	5.7 (0.0)	2.4 (0.0)	2.5 (0.0)	3.7 (0.0)	2.5 (0.0)	2.8 (0.0)	3.9 (0.0)	3.4 (0.0)	2.7 (0.0)	3.7 (0.0)
**Vitamin B_12_**, µg/day ^10^												
Naturally occurring	4.1 (0.0)	4 (0.0)	4.3 (0.0)	3.6 (0.0)	3.6 (0.0)	3.8 (0.0)	3.9 (0.0)	3.9 (0.0)	4.1 (0.0)	3.7 (0.0)	3.7 (0.0)	3.9 (0.0)
+ Enriched/fortified	5.4 (0.0)	5.2 (0.0)	5.5 (0.0)	4.6 (0.0)	4.4 (0.0)	4.7 (0.0)	5.0 (0.0)	4.9 (0.0)	5.2 (0.0)	4.7 (0.0)	4.6 (0.0)	4.9 (0.0)
+ Dietary supplements	37.5 (0.4)	41.8 (1.0)	45.3 (0.4)	13.7 (0.4)	15.2 (0.4)	32.4 (0.4)	10.8 (0.2)	14.7 (0.4)	31.8 (0.6)	22.5 (0.6)	21.8 (0.8)	37.6 (0.8)
**Folate**, µg DFE/day ^10,11^												
Naturally occurring	193 (0.2)	199 (0.2)	226 (0.2)	159 (0.2)	169 (0.2)	198 (0.2)	192 (0.2)	203 (0.4)	231 (0.2)	192 (0.4)	197 (0.6)	222 (0.2)
+ Enriched/fortified	533 (0.4)	531 (0.8)	569 (0.4)	458 (0.4)	464 (0.8)	497 (0.4)	536 (0.4)	542 (0.8)	578 (0.6)	550 (0.8)	553 (1.4)	589 (0.6)
+ Dietary supplements	671 (0.8)	710 (1.8)	815 (0.8)	538 (0.8)	585 (1.6)	639 (1.0)	603 (0.8)	627 (1.4)	710 (1.0)	664 (1.6)	688 (2.6)	765 (1.4)
**Thiamin**, mg/day ^10^												
Naturally occurring	0.9 (0.0)	0.9 (0.0)	1.0 (0.0)	0.8 (0.0)	0.8 (0.0)	0.9 (0.0)	0.9 (0.0)	0.9 (0.0)	1.0 (0.0)	0.9 (0.0)	0.9 (0.0)	1.0 (0.0)
+ Enriched/fortified	1.6 (0.0)	1.6 (0.0)	1.7 (0.0)	1.4 (0.0)	1.4 (0.0)	1.5 (0.0)	1.6 (0.0)	1.6 (0.0)	1.7 (0.0)	1.5 (0.0)	1.6 (0.0)	1.6 (0.0)
+ Dietary supplements	4.7 (0.2)	3.8 (0.0)	5.1 (0.0)	1.7 (0.0)	2.6 (0.0)	3.0 (0.0)	2.0 (0.0)	2.1 (0.0)	3.4 (0.0)	2.9 (0.0)	4.1 (0.2)	3.5 (0.0)
**Riboflavin**, mg/day ^10^												
Naturally occurring	1.5 (0.0)	1.6 (0.0)	1.7 (0.0)	1.1 (0.0)	1.2 (0.0)	1.3 (0.0)	1.4 (0.0)	1.4 (0.0)	1.5 (0.0)	1.3 (0.0)	1.4 (0.0)	1.5 (0.0)
+ Enriched/fortified	2.1 (0.0)	2.1 (0.0)	2.3 (0.0)	1.7 (0.0)	1.7 (0.0)	1.8 (0.0)	1.9 (0.0)	2.0 (0.0)	2.1 (0.0)	1.8 (0.0)	1.8 (0.0)	1.9 (0.0)
+ Dietary supplements	3.2 (0.0)	3.5 (0.0)	4.5 (0.0)	2.0 (0.0)	2.3 (0.0)	3.1 (0.0)	2.4 (0.0)	2.5 (0.0)	3.5 (0.0)	2.8 (0.0)	2.9 (0.0)	3.3 (0.0)
**Niacin**, mg/day ^10^												
Naturally occurring	16.4 (0.0)	16.4 (0.0)	18.2 (0.0)	15.4 (0.0)	15.9 (0.0)	18.1 (0.0)	15.5 (0.0)	16.2 (0.0)	18.0 (0.0)	15.5 (0.0)	15.6 (0.0)	17.3 (0.0)
+ Enriched/fortified	23.7 (0.0)	23.3 (0.0)	25.3 (0.0)	21.9 (0.0)	22.0 (0.0)	24.3 (0.0)	22.7 (0.0)	23.1 (0.0)	25.1 (0.0)	22.2 (0.0)	22.1 (0.0)	23.9 (0.0)
+ Dietary supplements	28.6 (0.0)	29.1 (0.0)	37.3 (0.2)	24.0 (0.0)	24.8 (0.0)	31.6 (0.2)	24.4 (0.0)	25.2 (0.0)	30.1 (0.0)	25 (0.0)	25.8 (0.0)	30.2 (0.2)
**Iron**, mg/day ^5^												
Naturally occurring	8.7 (0.0)	8.7 (0.0)	9.6 (0.0)	7.8 (0.0)	8.0 (0.0)	9.0 (0.0)	8.5 (0.0)	8.8 (0.0)	9.7 (0.0)	8.3 (0.0)	8.4 (0.0)	9.1 (0.0)
+ Enriched/fortified	14.8 (0.0)	14.7 (0.0)	15.6 (0.0)	13.2 (0.0)	13.3 (0.0)	14.2 (0.0)	14.8 (0.0)	14.8 (0.0)	15.8 (0.0)	14.1 (0.0)	14 (0.0)	14.9 (0.0)
+ Dietary supplements	17.3 (0.0)	17.9 (0.0)	19.4 (0.0)	15.6 (0.0)	16.6 (0.0)	16.8 (0.0)	16.6 (0.0)	16.2 (0.0)	18.0 (0.0)	15.5 (0.0)	17.4 (0.2)	17.0 (0.0)
**Zinc**, mg/day ^5^												
Naturally occurring	9.8 (0.0)	9.7 (0.0)	10.6 (0.0)	8.7 (0.0)	8.7 (0.0)	9.7 (0.0)	9.6 (0.0)	9.8 (0.0)	10.6 (0.0)	9.2 (0.0)	9.2 (0.0)	10.0 (0.0)
+ Enriched/fortified	11.0 (0.0)	10.9 (0.0)	11.8 (0.0)	9.6 (0.0)	9.6 (0.0)	10.5 (0.0)	10.6 (0.0)	10.7 (0.0)	11.5 (0.0)	10.2 (0.0)	10.1 (0.0)	10.9 (0.0)
+ Dietary supplements	13.4 (0.0)	14.0 (0.0)	16.4 (0.0)	10.9 (0.0)	11.5 (0.0)	13.1 (0.0)	11.7 (0.0)	11.9 (0.0)	13.9 (0.0)	11.7 (0.0)	12 (0.0)	14.0 (0.0)
**Calcium**, mg/day ^7,8^												
Naturally occurring	960 (0.8)	946 (1.2)	999 (0.6)	770 (0.6)	763 (1.2)	800 (0.8)	933 (0.6)	932 (1.2)	977 (0.8)	791 (1.2)	801 (1.8)	847 (1.0)
+ Enriched/fortified	1036 (0.8)	1024 (1.4)	1079 (0.6)	842 (0.8)	836 (1.4)	872 (0.8)	1015 (0.8)	1013 (1.4)	1062 (1.0)	858 (1.4)	867 (2.2)	918 (1.0)
+ Dietary supplements	1130 (1.0)	1158 (1.8)	1258 (0.8)	878 (0.8)	911 (1.6)	963 (1.0)	1054 (0.8)	1060 (1.4)	1150 (1.2)	920 (1.6)	918 (2.4)	1018 (1.4)
**Magnesium**, mg/day ^8^												
Naturally occurring	266 (0.2)	273 (0.4)	303 (0.2)	224 (0.2)	235 (0.4)	266 (0.2)	262 (0.2)	275 (0.4)	305 (0.2)	256 (0.4)	263 (0.6)	290 (0.4)
+ Enriched/fortified	270 (0.2)	277 (0.4)	307 (0.2)	226 (0.2)	237 (0.4)	269 (0.2)	264 (0.2)	277 (0.4)	308 (0.2)	258 (0.4)	264 (0.6)	292 (0.4)
+ Dietary supplements	284 (0.2)	298 (0.6)	337 (0.2)	231 (0.2)	246 (0.4)	286 (0.4)	271 (0.2)	282 (0.4)	322 (0.4)	266 (0.4)	272 (0.6)	307 (0.4)
**Potassium**, g/day ^12,13^												
Naturally occurring	2476 (1.8)	2520 (3.0)	2755 (1.4)	2089 (1.8)	2165 (3.0)	2412 (2.0)	2429 (1.6)	2511 (3.0)	2746 (2.2)	2320 (3.2)	2354 (5)	2561 (2.6)
+ Enriched/fortified	2494 (1.8)	2535 (3.0)	2775 (1.4)	2101 (1.8)	2174 (3.0)	2426 (2.0)	2439 (1.6)	2517 (3.0)	2758 (2.2)	2336 (3.2)	2366 (5)	2579 (2.6)
+ Dietary supplements	2502 (1.8)	2547 (3.0)	2793 (1.4)	2104 (1.8)	2180 (3.0)	2434 (2.0)	2442 (1.6)	2520 (3.0)	2768 (2.2)	2341 (3.2)	2370 (5)	2586 (2.6)

^1^ Mean ± standard error.^2^ Data Source: What We Eat in America, NHANES 2009–2012 [17]. ^3^ Usual intake distribution estimated using the National Cancer Institute Method for individuals 2 years of age and older, including pregnant and lactating women. Accessible via https://epi.grants.cancer.gov/diet/usualintakes/method.html. ^4^ The other race/ethnicity group included non-Hispanic persons reporting multiple races. ^5^ Dietary reference intakes for Vitamin A, Vitamin K, Arsenic, Boron, Chromium, Copper, Iodine, Iron, Manganese, Molybdenum, Nickel, Silicon, Vanadium, and Zinc (2001) [21]. ^6^ A supplements file is not currently available for Vitamins A and E in NHANES for 2009–2012, and it will be released at a later date. ^7^ Dietary reference intakes for calcium and vitamin D (2011) [27]. ^8^ Dietary reference intakes for calcium, phosphorous, magnesium, vitamin D, and fluoride (1997) [22]. ^9^ Dietary reference intakes for vitamin C, vitamin E, selenium, and carotenoids (2000) [28]. ^10^ Dietary reference intakes for thiamin, riboflavin, niacin, vitamin B_6_, folate, vitamin B_12_, pantothenic acid, biotin, and choline (1998) [19]. ^11^ Folate EAR is presented as dietary folate equivalents (DFE). 1 DFE = 1 µg food folate = 0.6 µg of folic acid from fortified food or supplement consumed with food = 5 µg of a supplement taken on an empty stomach. ^12^ Dietary reference intakes for water, potassium, sodium, chloride, and sulfate (2005) [20]. ^13^ The AI approach was used for potassium. U.S., United States. NHANES, National Health and Nutrition Examination Survey. PIR, poverty index ratio. RAE, retinol activity equivalents. AT, a-tocopherol. DFE, dietary folate equivalents. The shading present differentiate between the three PIR categories.

**Table 3:** Percent less than the estimated average requirement for the U.S. population 2 years of age and older by race/ethnicity and the poverty index ratio category, NHANES 2009–2012 ^1–4^.

	Non-Hispanic White			Non-Hispanic Black			Hispanic			Other Race/Ethnicity ^5^		
	PIR Category			PIR Category			PIR Category			PIR Category		
Nutrient	<131%	131–185%	>185%	<131%	131–185%	>185%	<131%	131–185%	>185%	<131%	131–185%	>185%
**Vitamin A**, µg RAE/day ^6,7^												
Naturally occurring	70.4 (1.8)	69.4 (2.4)	62.9 (2.0)	**75.0 (1.8)**	**77.8 (2.4)**	**75.4 (2.6)**	**66.3 (1.6)**	69.5 (2.4)	61.9 (1.8)	69.5 (1.8)	68.3 (2.8) *	**59.1 (2.4)**
+ Enriched/fortified	38.1 (1.8)	36.6 (2.8)	29.7 (1.6)	**49.7 (2.4)**	**52.0 (3.2)**	**48.8 (3.0)**	**39.5 (1.6)**	**42.8 (2.2)**	**34.8 (1.6)**	**43.9 (2.0)**	**43.3 (3.0)**	**34.0 (2.0)**
+ Dietary supplements	--	--	--	--	--	--	--	--	--	--	--	--
**Vitamin D**, µg/day ^8,9^												
Naturally occurring	100 (0.0)	100 (0.0)	100 (0.0)	100 (0.0)	100 (0.0)	100 (0.0)	100 (0.0)	100 (0.0)	100 (0.0)	100 (0.0)	100 (0.0)	100 (0.0)
+ Enriched/fortified	92.7 (0.8)	93.5 (0.8)	93.2 (0.8)	**96.1 (0.6)**	**96.8 (0.6)**	**97.3 (0.4)**	**90.1 (0.8)**	**92.1 (1.2)**	**91.7 (0.8)**	**94.2 (1.0)**	**94.6 (1.2)**	**94.1 (1.4)**
+ Dietary supplements	73.1 (0.6)	67.0 (0.6)	59.7 (0.6)	**84.4 (0.6)**	**80.1 (0.6)**	**75.4 (0.4)**	**81.4 (0.6)**	**81.8 (1.0)**	**73.9 (0.8)**	**77.4 (0.8)**	**72.4 (1.0)**	**67.5 (1.0)**
**Vitamin E**, mg AT/day ^7,10^												
Naturally occurring	90.1 (0.8)	89.8 (1.0)	84.4 (0.8)	88.9 (0.8)	90.0 (0.8)	**86.9 (0.8)**	87.9 (1.2)	89.1 (1.2)	**83.1 (1.8)**	90.7 (1.2)	89.6 (1.6)	83.0 (1.2)*
+ Enriched/fortified	85.3 (1.0)	85.2 (1.4)	77.1 (0.8)	85.0 (0.8)	**86.5 (1.2)**	**81.7 (0.8)**	**83.7 (1.4)**	85.2 (1.2) *	77.1 (2.0) *	**86.6 (1.6)**	85.2 (2.0)*	**76.1 (1.6)**
+ Dietary supplements	--	--	--	--	--	--	--	--	--	--	--	--
**Vitamin C**, mg/day ^10^												
Naturally occurring	62.6 (2.0)	58.3 (3.2)	50.3 (1.4)	**58.6 (1.8)**	57.4 (3.2)	52.4 (2.0)	**44.9 (1.8)**	**44.8 (3.0)**	**35.3 (2.2)**	**52.9 (3.0)**	**49.4 (3.6)**	**39.1 (2.8)**
+ Enriched/fortified	44.5 (1.8)	42.8 (2.6)	36.3 (0.8)	**32.8 (1.6)**	**33.4 (3.2)**	**30.2 (2.0)**	**26.4 (1.8)**	**28.3 (2.4)**	**22.0 (1.8)**	**36.0 (2.2)**	**34.9 (3.0)**	**27.4 (2.4)**
+ Dietary supplements	36.0 (1.4)	32.8 (2.0)	24.1 (0.6)	**29.2 (1.4)**	**28.8 (2.6)**	**24.0 (1.6)**	**23.9 (1.6)**	**25.4 (2.2)**	**18.0 (1.4)**	**30.6 (2.0)**	**27.5 (2.4)**	**20.0 (1.6)**
**Vitamin B_6_**, mg/day ^11^												
Naturally occurring	19.9 (1.4)	21.8 (1.8)	15.8 (0.8)	19.1 (1.2)	21.4 (1.6)	15.9 (1.2) *	**11.7 (1.4)**	**12.4 (1.0)**	**8.2 (1.4)**	**15.0 (1.4)**	**14.1 (1.8)**	**8.4 (1.0)**
+ Enriched/fortified	8.6 (1.0)	10.4 (1.0)	7.2 (0.6)	**9.9 (1.0)**	**12.7 (1.0)**	**9.3 (0.8)**	**5.3 (0.8)**	**6.4 (0.8)**	**4.0 (0.8)**	8.2 (1.0) *	**8.2 (1.2)**	**4.8 (0.6)**
+ Dietary supplements	6.7 (0.8)	7.2 (0.8)	4.3 (0.4)	**8.6 (0.8)**	**10.7 (1.0)**	**7.0 (0.6)**	**4.7 (0.8)**	**5.6 (0.8)**	**3.2 (0.6)**	6.7 (0.8)	**6.4 (1.0)**	**3.3 (0.4)**
**Vitamin B_12_**, µg/day ^11^												
Naturally occurring	4.4 (0.6)	5.2 (0.6)	3.8 (0.4)	**6.8 (0.8)**	**8.0 (1.0)**	**6.8 (0.8)**	4.5 (0.6) *	5.5 (1.0) *	**4.1 (0.6)**	**6.2 (0.8)**	**6.6 (1.0)**	**4.7 (0.8)**
+ Enriched/fortified	1.8 (0.2)	2.3 (0.4)	1.6 (0.2)	**3.6 (0.6)**	**4.7 (0.6)**	**3.9 (0.6)**	**2.1 (0.4)**	**2.9 (0.6)**	**2.0 (0.4)**	**3.3 (0.4)**	**3.7 (0.6)**	**2.6 (0.4)**
+ Dietary supplements	1.4 (0.2)	1.7 (0.2)	1.0 (0.2)	**3.2 (0.4)**	**4.0 (0.6)**	**3.0 (0.4)**	**1.9 (0.2)**	**2.6 (0.4)**	**1.7 (0.2)**	**2.6 (0.4)**	**2.9 (0.6)**	**1.9 (0.2)**
**Folate**, µg DFE/day ^11,12^												
Naturally occurring	86.5 (1.0)	86.4 (1.2)	80.2 (1.2)	**89.8 (0.6)**	**91.1 (0.8)**	**88.1 (1.0)**	**79.7 (1.6)**	**81.3 (1.4)**	**73.3 (2.4)**	**83.5 (1.8)**	**82.7 (2.6)**	**74.3 (2.0)**
+ Enriched/fortified	8.3 (1.0)	9.1 (1.0)	6.7 (0.6)	**13.5 (1.4)**	**14.7 (1.8)**	**12.7 (1.2)**	**6.3 (0.8)**	**7.3 (1.0)**	**5.0 (0.8)**	**6.4 (1.4)**	**6.4 (1.4)**	**4.4 (1.0)**
+ Dietary supplements	6.6 (0.8)	6.7 (0.8)	4.3 (0.4)	**11.9 (1.2)**	**12.6 (1.6)**	**10.0 (1.0)**	**5.6 (0.8)**	6.3 (1.0)	4.1 (0.6)	**5.3 (1.2)**	**5.0 (1.2)**	**3.1 (0.6)**
**Thiamin**, mg/day ^11^												
Naturally occurring	45.8 (1.8)	45.9 (2.8)	38.2 (1.4)	**55.8 (2.0)**	**57.8 (2.2)**	**53.6 (1.6)**	**42.4 (1.8)**	44.6 (2.2)	36.5 (2.0)	46.8 (3.2) *	45.3 (4) *	**35.6 (3.0)**
+ Enriched/fortified	5.4 (0.6)	6.0 (0.6)	4.5 (0.4)	**8.5 (0.8)**	**9.8 (0.8)**	**8.3 (0.6)**	**4.6 (0.6)**	**5.3 (0.6)**	**3.8 (0.6)**	**5.7 (1.0)**	5.3 (1.0) *	**3.8 (0.6)**
+ Dietary supplements	4.4 (0.4)	4.4 (0.4)	2.9 (0.2)	**7.6 (0.8)**	**8.4 (0.8)**	**6.5 (0.6)**	4.1 (0.4)	**4.7 (0.6)**	**3.2 (0.4)**	**4.9 (0.8)**	4.2 (0.8) *	2.8 (0.4)
**Riboflavin**, mg/day ^11^												
Naturally occurring	12.9 (1.2)	12 (1.4)	8.9 (0.6)	**28.1 (1.6)**	**28.7 (2.0)**	**24.4 (1.2)**	**15.3 (1.2)**	**15.8 (1.4)**	**11.4 (1.2)**	**20.4 (1.8)**	**19.0 (2.4)**	**14.0 (1.6)**
+ Enriched/fortified	2.3 (0.2)	2.5 (0.4)	1.6 (0.2)	**7.6 (0.8)**	**8.6 (1.0)**	**6.8 (0.8)**	**3.3 (0.4)**	**3.8 (0.6)**	**2.5 (0.4)**	**6.2 (0.8)**	**6.0 (1.0)**	**4.0 (0.6)**
+ Dietary supplements	1.9 (0.2)	1.9 (0.4)	1.1 (0.2)	**7.0 (0.8)**	**7.5 (1.0)**	**5.5 (0.6)**	**3.1 (0.4)**	**3.5 (0.6)**	**2.2 (0.4)**	**5.4 (0.8)**	**5.1 (0.8)**	**3.0 (0.4)**
**Niacin**, mg/day ^11^												
Naturally occurring	11.4 (1.0)	12.5 (1.4)	7.6 (0.8)	11.4 (1.2)*	12.5 (1.4)*	7.5 (0.8)	10.4 (1.2) *	**10.8 (1.0)**	**6.1 (1.0)**	**12.8 (1.6)**	12.5 (2.0)	**7.0 (1.0)**
+ Enriched/fortified	1.5 (0.2)	2.0 (0.4)	1.1 (0.2)	**1.7 (0.4)**	**2.4 (0.4)**	**1.4 (0.2)**	**1.2 (0.2)**	**1.5 (0.2)**	**0.8 (0.2)**	**2.0 (0.4)**	1.9 (0.4)	1.1 (0.2)*
+ Dietary supplements	1.2 (0.2)	1.4 (0.2)	0.7 (0.2)	**1.5 (0.2)**	**2.0 (0.2)**	**1.1 (0.2)**	1.1 (0.2)	1.3 (0.2)	0.7 (0.2)	**1.7 (0.2)**	**1.5 (0.4)**	0.8 (0.2)
**Iron**, mg/day ^6^												
Naturally occurring	16.3 (1.2)	15.8 (1.8)	10.4 (0.8)	**23.5 (1.4)**	**21.5 (1.8)**	**15.0 (1.0)**	15.5 (1.4)	**16.4 (1.6)**	9.5 (1.0)*	**19.1 (2.2)**	**20.5 (2.6)**	**13.6 (1.6)**
+ Enriched/fortified	1.7 (0.2)	1.8 (0.2)	1.5 (0.2)	**3.6 (0.4)**	**3.1 (0.4)**	**2.2 (0.2)**	1.8 (0.2)*	**2.5 (0.2)**	**1.0 (0.2)**	**2.1 (0.4)**	**2.9 (0.4)**	**2.2 (0.2)**
+ Dietary supplements	1.4 (0.2)	1.6 (0.2)	0.9 (0.2)	**3.1 (0.4)**	**2.7 (0.4)**	**1.8 (0.2)**	1.4 (0.2)*	**1.7 (0.2)**	0.9 (0.2)*	**2.1 (0.4)**	**1.8 (0.4)**	**1.3 (0.2)**
**Zinc**, mg/day ^6^												
Naturally occurring	22.9 (1.4)	25.3 (2.2)	19.8 (1.2)	**27.7 (1.6)**	**33.6 (2.0)**	**27.1 (1.6)**	**19.0 (1.4)**	**22.4 (1.8)**	**16.4 (1.2)**	**26.8 (2.2)**	**26.8 (2.6)**	19.8 (1.8)
+ Enriched/fortified	16.3 (1.2)	18.2 (1.8)	14.7 (1.0)	**21.7 (1.6)**	**26.8 (1.8)**	**22.4 (1.6)**	**14.5 (1.0)**	17.6 (1.4)	**13.6 (0.8)**	**20.8 (1.8)**	**21.0 (2.0)**	**15.8 (1.4)**
+ Dietary supplements	13.4 (1.0)	13.8 (1.4)	9.8 (0.6)	**19.7 (1.4)**	**23.4 (1.6)**	**17.9 (1.2)**	13.3 (1.0)	**16.2 (1.4)**	**11.4 (0.6)**	**18.0 (1.6)**	**18.1 (1.6)**	**11.8 (1.0)**
**Calcium**, mg/day ^8,9^												
Naturally occurring	43.6 (1.4)	46.1 (2.6)	41.9 (0.8)	**65.1 (1.8)**	**67.7 (2.6)**	**64.3 (1.6)**	**47.0 (1.6)**	46.9 (3.2) *	42.3 (2.0) *	**62.1 (2.0)**	**62.2 (3.8)**	**55.3 (2.4)**
+ Enriched/fortified	36.8 (1.2)	39.0 (2.6)	34.9 (0.8)	**56.9 (1.8)**	**59.5 (2.8)**	**56.2 (1.8)**	**39.3 (1.6)**	38.9 (2.6)	34.8 (2.0)	**54.6 (2.6)**	**55.0 (4.6)**	**47.7 (2.8)**
+ Dietary supplements	30.4 (1.2)	29.5 (2.0)	24.5 (0.6)	**53.3 (1.8)**	**52.9 (2.6)**	**48.3 (1.6)**	**36.4 (1.4)**	**35.5 (2.4)**	**29.1 (1.6)**	**48.3 (2.4)**	**49.6 (4.4)**	**39.5 (2.4)**
**Magnesium**, mg/day ^9^												
Naturally occurring	51.0 (1.6)	51.1 (2.2)	43.9 (1.0)	**55.7 (1.2)**	**60.2 (2.2)**	**57.2 (1.6)**	**41.8 (1.6)**	**45.6 (2.0)**	**36.9 (2.2)**	50.9 (2.2)	**50.1 (3.8)**	**39.2 (1.8)**
+ Enriched/fortified	49.7 (1.6)	50.0 (2.4)	42.5 (1.0)	**55.1 (1.2)**	**59.8 (2.2)**	**56.4 (1.6)**	**41.1 (1.4)**	**45.1 (2.0)**	**36.1 (2.2)**	50.2 (2.2)	**49.6 (3.8)**	**38.4 (1.8)**
+ Dietary supplements	45.4 (1.6)	44.4 (2.2)	35.4 (0.8)	**53.3 (1.2)**	**56.6 (2.2)**	**51.3 (1.6)**	**39.5 (1.4)**	43.4 (2.0)	**32.6 (2.0)**	**47.5 (2.2)**	46.6 (3.4) *	**34.6 (1.8)**
**Potassium**, g/day ^13,14^	**% >AI**			**% >AI**			**% >AI**			**% >AI**		
Naturally occurring	1.4 (0.2)	1.5 (0.2)	2.6 (0.4)	**0.6 (0.0)**	**0.6 (0.2)**	**0.9 (0.2)**	**2.0 (0.2)**	**1.8 (0.2)**	**3.1 (0.4)**	**1.1 (0.2)**	**1.3 (0.4)**	2.2 (0.6)*
+ Enriched/fortified	1.5 (0.2)	1.5 (0.4)	2.7 (0.4)	**0.6 (0.0)**	**0.6 (0.2)**	**1.0 (0.2)**	**2.0 (0.2)**	**1.9 (0.4)**	**3.2 (0.4)**	**1.2 (0.2)**	**1.3 (0.4)**	**2.3 (0.6)**
+ Dietary supplements	1.5 (0.2)	1.6 (0.4)	2.8 (0.4)	**0.6 (0.0)**	**0.6 (0.2)**	**1.0 (0.2)**	**2.0 (0.2)**	**1.9 (0.4)**	**3.3 (0.4)**	**1.2 (0.2)**	**1.3 (0.4)**	**2.3 (0.6)**

^1^ Average percent (standard error). ^2^ Statistical tests evaluating differences within race/ethnicity categories were run (Non-Hispanic white reference). Three regression models tested for race/ethnicity differences within PIR categories for each food/beverage level. Bold font signifies *p*-value < 0.0001. * signifies *p*-value < 0.05. ^3^ Data source: What We Eat in America, NHANES 2009–2012 [17]. ^4^ Usual intake distribution estimated using the National Cancer Institute Method for individuals two years of age and older, including pregnant and lactating women. Accessible via https://epi.grants.cancer.gov/diet/usualintakes/method.html. ^5^ The other race/ethnicity group included non-Hispanic persons reporting multiple races. ^6^ Dietary Reference Intakes for vitamin A, vitamin K, arsenic, boron, chromium, copper, iodine, iron, manganese, molybdenum, nickel, silicon, vanadium, and zinc (2001) [21]. ^7^ A supplemental file is not currently available for vitamins A and E in NHANES for 2009–2012, and it will be released at a later date. ^8^ Dietary reference intakes for calcium and vitamin D (2011) [27]. ^9^ Dietary reference intakes for calcium, phosphorous, magnesium, vitamin D, and fluoride (1997) [22]. ^10^ Dietary reference intakes for vitamin C, vitamin E, selenium, and carotenoids (2000) [28]. ^11^ Dietary reference intakes for thiamin, riboflavin, niacin, vitamin B_6_, folate, vitamin B_12_, pantothenic acid, biotin, and choline (1998) [19]. ^12^ Folate EAR is presented as dietary folate equivalents (DFE). 1 DFE = 1 µg food folate = 0.6 µg of folic acid from fortified food or supplement consumed with food = 5 µg of a supplement taken on an empty stomach. ^13^ Dietary reference intakes for water, potassium, sodium, chloride, and sulfate (2005) [20]. ^14^ The AI approach was used for potassium. U.S., United States. NHANES, National Health and Nutrition Examination Survey. PIR, poverty index ratio. RAE, retinol activity equivalents. AT, a-tocopherol. DFE, dietary folate equivalents. The shading present differentiate between the three PIR categories.

**Table 4:** Percent greater than or equal to the tolerable upper intake level for the U.S. population two years of age and older by race/ethnicity and the poverty index ratio category, NHANES 2009–012 ^1–4^.

	Non-Hispanic White			Non-Hispanic Black			Hispanic			Other Race/Ethnicity ^5^		
	PIR Category			PIR Category			PIR Category			PIR Category		
Nutrient ^6^	<131%	131–185%	>185%	<131%	131–185%	>185%	<131%	131–185%	>185%	<131%	131–185%	>185%
**Vitamin A**, µg RAE/day ^6,7^												
Naturally occurring	0.6 (0.2)	0.7 (0.2)	0.8 (0.2)	**0.4 (0.2)**	**0.4 (0.0)**	**0.4 (0.0)**	**0.9 (0.2)**	0.7 (0.2)	**1.0 (0.2)**	**0.8 (0.2)**	**1.0 (0.2)**	**1.4 (0.4)**
+ Enriched/fortified	4.8 (0.2)	4.5 (0.4)	4.2 (0.2)	**4.0 (0.6)**	**2.9 (0.4)**	**2.6 (0.2)**	**6.3 (0.6)**	**4.8 (0.6)**	**5.5 (0.4)**	**5.2 (0.4)**	**5.5 (0.6)**	**7.0 (0.6)**
**Vitamin D**, µg/day ^8–10^												
+ Dietary supplements	0.4 (0.0)	0.5 (0.0)	1.0 (0.0)	**0.1 (0.0)**	**0.2 (0.0)**	1.0 (0.0)	**0.1 (0.0)**	**0.0 (0.0)**	**0.3 (0.0)**	0.2 (0.0)*	0.5 (0.0)	**0.9 (0.0)**
**Vitamin C**, mg/day ^8,11^												
+ Dietary supplements	0.4 (0.0)	0 (0.0)	0.6 (0.0)	**0.1 (0.0)**	0.2 (0.0)*	**0.4 (0.0)**	**0.0 (0.0)**	0.0 (0.0)	**0.4 (0.0)**	0.2 (0.0)	**1.4 (0.0)**	**0.7 (0.0)**
**Vitamin B_6_**, mg/day ^8,12^												
+ Dietary supplements	0.5 (0.0)	0.8 (0.0)	1.1 (0.0)	**0.2 (0.0)**	**0.2 (0.0)**	**0.3 (0.0)**	**0.2 (0.0)**	**0.3 (0.0)**	**0.4 (0.0)**	**0.6 (0.0)**	**0 (0.0)**	**0.2 (0.0)**
**Folate**, µg DFE/day ^12,13^												
Naturally occurring	0.1 (0.0)	0.1 (0.0)	0.1 (0.0)	**0.0 (0.0)**	**0.0 (0.0)**	0.1 (0.0)	**0.2 (0.0)**	**0.2 (0.0)**	**0.3 (0.0)**	0.1 (0.0)	**0.2 (0.0)**	**0.3 (0.0)**
+ Enriched/fortified	16.7 (0.8)	14.3 (0.8)	14.8 (0.8)	**18.3 (0.8)**	**13.7 (1.0)**	**10.5 (0.6)**	**27.1 (1.0)**	**20.8 (1.2)**	**22.0 (1.0)**	**22.4 (1.4)**	**22.6 (1.8)**	**25.6 (1.4)**
+ Dietary supplements	30.4 (0.8)	32.0 (0.8)	39.7 (0.6)	**25.5 (0.8)**	**24.4 (0.8)**	**25.1 (0.6)**	**33.1 (1.0)**	**27.9 (1.2)**	**34.8 (0.8)**	**32.8 (1.4)**	**34.9 (1.6)**	**42.6 (1.2)**
**Niacin**, mg/day ^12^												
+ Enriched/fortified	1.9 (0.2)	1.5 (0.2)	1.3 (0.2)	**2.0 (0.4)**	**1.3 (0.2)**	**0.9 (0.2)**	**3.0 (0.2)**	**2.0 (0.2)**	**1.8 (0.2)**	1.9 (0.4)	**2.0 (0.2)**	**1.9 (0.2)**
+ Dietary supplements	7.2 (0.2)	6.8 (0.2)	9.3 (0.2)	5.1 (0.4)*	**5.9 (0.2)**	**5.4 (0.8)**	**4.9 (0.2)**	**5.2 (0.2)**	**7.3 (0.2)**	**5.6 (0.4)**	7.2 (0.2)	**7.5 (0.2)**
**Iron**, mg/day ^7,8^												
+ Dietary supplements	1.9 (0.0)	2.3 (0.0)	2.6 (0.0)	**2.0 (0.0)**	**2.5 (0.0)**	**2.1 (0.0)**	**1.6 (0.0)**	**1.2 (0.0)**	**2.0 (0.0)**	**0.9 (0.0)**	**1.6 (0.0)**	**1.3 (0.0)**
**Zinc**, mg/day ^7^												
Naturally occurring	4.0 (0.2)	3.5 (0.2)	3.2 (0.2)	**4.7 (0.2)**	**3.1 (0.2)**	**2.7 (0.2)**	**6.9 (0.4)**	**4.8 (0.4)**	**5.1 (0.2)**	**4.7 (0.4)**	**4.8 (0.4)**	**5.7 (0.4)**
+ Enriched/fortified	6.3 (0.2)	5.4 (0.4)	4.7 (0.2)	**7.3 (0.4)**	**4.8 (0.4)**	**3.8 (0.2)**	**10.1 (0.4)**	**7.1 (0.4)**	**7.2 (0.4)**	**7.2 (0.4)**	**7.0 (0.6)**	**8.1 (0.4)**
+ Dietary supplements	8.8 (0.2)	8.1 (0.4)	8.8 (0.2)	**9.2 (0.4)**	**7.1 (0.4)**	**5.4 (0.2)**	**11.3 (0.4)**	**9.0 (0.4)**	**9.9 (0.4)**	**9.3 (0.4)**	**8.9 (0.6)**	**12 (0.4)**
**Calcium**, mg/day ^9,10^												
Naturally occurring	0.1 (0.0)	0.1 (0.0)	0.2 (0.0)	**0.0 (0.0)**	**0.0 (0.0)**	**0.0 (0.0)**	**0.0 (0.0)**	0.0 (0.0)	0.1 (0.0)	**0.0 (0.0)**	**0.0 (0.0)**	**0.0 (0.0)**
+ Enriched/fortified	0.3 (0.0)	0.3 (0.0)	0.5 (0.0)	**0.0 (0.0)**	**0.0 (0.0)**	**0.1 (0.0)**	**0.1 (0.0)**	**0.1 (0.0)**	**0.3 (0.0)**	**0.0 (0.0)**	**0.0 (0.0)**	**0.1 (0.0)**
+ Dietary supplements	2.1 (0.0)	3.5 (0.4)	5.6 (0.2)	**0.3 (0.0)**	**1.2 (0.0)**	**1.5 (0.0)**	**0.8 (0.0)**	**0.9 (0.2)**	**2.5 (0.2)**	**0.5 (0.0)**	**0.2 (0.0)**	**1.4 (0.2)**
**Magnesium**, mg/day ^8,10,14^												
+ Dietary supplements	0.5 (0.0)	0.9 (0.0)	1.5 (0.0)	**0.1 (0.0)**	**0.2 (0.0)**	**0.8 (0.0)**	**0.3 (0.0)**	**0.1 (0.0)**	**0.6 (0.0)**	**0.4 (0.0)**	**0.0 (0.0)**	**0.4 (0.0)**

^1^ Average percent (standard error). ^2^ Statistical tests evaluating differences within race/ethnicity categories were run (Non-Hispanic white reference). Three regression models tested for race/ethnicity differences within PIR categories for each food/beverage level. Bold font signifies *p*-value < 0.0001. * signifies *p*-value <0.05. ^3^ Data source: What We Eat in America, NHANES 2009–2012 [17]. ^4^ Usual intake distribution estimated using the National Cancer Institute Method for individuals two years of age and older, including pregnant and lactating women. Accessible via https://epi.grants.cancer.gov/diet/usualintakes/method.html. ^5^ The other race/ethnicity group included non-Hispanic persons reporting multiple races. ^6^ A UL was not available for the following nutrients: Vitamin A (retinol activity equivalents [RAE]) and vitamin E (a-tocopherol [AT]) for supplements, vitamin B_12_, thiamin, riboflavin, and potassium for all levels.^7^ Dietary reference intakes for vitamin A, vitamin K, arsenic, boron, chromium, copper, iodine, iron, manganese, molybdenum, nickel, silicon, vanadium, and zinc (2001) [21]. ^8^ Values were zero until dietary supplements were added. ^9^ Dietary reference intakes for calcium and vitamin D (2011) [27]. ^10^ Dietary reference intakes for calcium, phosphorous, magnesium, vitamin D, and fluoride (1997) [22]. ^11^ Dietary reference intakes for vitamin C, vitamin E, selenium, and carotenoids (2000) [28]. ^12^ Dietary reference intakes for thiamin, riboflavin, niacin, vitamin B_6_, folate, vitamin B_12_, pantothenic acid, biotin, and choline (1998) [19]. ^13^ Folate EAR is presented as dietary folate equivalents (DFE). 1 DFE = 1 µg food folate = 0.6 µg of folic acid from fortified food or a supplement consumed with food = 5 µg of a supplement taken on an empty stomach. ^14^ UL for magnesium and niacin established only for supplemental sources. U.S., United States. NHANES, National Health and Nutrition Examination Survey. PIR, poverty index ratio. DFE, dietary folate equivalents. The shading present differentiate between the three PIR categories.

## Supplementary Materials

The following are available online at https://www.mdpi.com/2072-6643/11/5/1005/s1. Table S1: Descriptive Statistics of Sample Size for Interview Day 1 and Day 2 for the U.S. Population 2 Years of Age and Older, NHANES 2009–2012.

## References

[1] US Department of Health and Human Services and USDA 2015–2020 Dietary Guidelines for Americans. 2015; eighth edition. https://health.gov/dietaryguidelines/2015/guidelines/.

[2] US Department of Health and Human Services and USDA (2015). Scientific Report of the 2015 Dietary Guidelines Advisory Committee. https://ods.od.nih.gov/pubs/2015_DGAC_Scientific_Report.pdf.

[3] Backstrand J.R. (2002). The history and future of food fortification in the United States: A public health perspective. Nutr. Rev..

[4] Dwyer J., Woteki C., Bailey R.L., Brittten P., Carriquiry A., Gaine P.C., Miller D., Moshfegh A., Murphy M.M., Edge M.S. (2014). Fortification: New findings and implications. Nutr. Rev..

[5] Fulgoni V.L., Keast D.R., Bailey R.L., Dwyer J. (2011). Foods, fortificants, and supplements: Where do Americans get their nutrients?. J. Nutr..

[6] Wang Y., Chen X. (2011). How much of racial/ethnic disparities in dietary intakes, exercise, and weight status can be explained by nutrition- and health-related psychosocial factors and socioeconomic status among US adults?. J. Am. Diet Assoc..

[7] Blumberg J.B., Frei B., Fulgoni V.L., Weaver C.M., Zeisel S.H. (2018). Contribution of Dietary Supplements to Nutritional Adequacy by Socioeconomic Subgroups in Adults of the United States. Nutrients.

[8] Bailey R.L., Akabas S.R., Paxson E.E., Thuppal S.V., Saklani S., Tucker K.L. (2017). Total Usual Intake of Shortfall Nutrients Varies With Poverty Among US Adults. J. Nutr. Educ. Behav..

[9] Storey M.L., Anderson P.A. (2016). Vegetable Consumption and Selected Nutrient Intakes of Women of Childbearing Age. J. Nutr. Educ. Behav..

[10] Blumberg J.B., Frei B., Fulgoni V.L., Weaver C.M., Zeisel S.H. (2017). Contribution of Dietary Supplements to Nutritional Adequacy in Race/Ethnic Population Subgroups in the United States. Nutrients.

[11] Berner L.A., Keast D.R., Bailey R.L., Dwyer J.T. (2014). Fortified foods are major contributors to nutrient intakes in diets of US children and adolescents. J. Acad. Nutr. Diet.

[12] Berner L.A., Clydesdale F.M., Douglass J.S. (2001). Fortification contributed greatly to vitamin and mineral intakes in the United States, 1989–1991. J. Nutr..

[13] Bailey R.L., Gahche J.J., Lentino C.V., Dwyer J.T., Engel J.S., Thomas P.R., Betz J.M., Sempos C.T., Picciano M.F. (2011). Dietary supplement use in the United States, 2003–2006. J. Nutr..

[14] Dodd K.W., Guenther P.M., Freedman L.S., Subar A.F., Kipnis V., Midthune D., Tooze J.A., Krebs-Smith S.M. (2006). Statistical methods for estimating usual intake of nutrients and foods: A review of the theory. J. Am Diet Assoc..

[15] Tooze J.A., Midthune D., Dodd K.W., Freedman L.S., Krebs-Smith S.M., Subar A.F., Guenther P.M., Carroll R.J., Kipnis V. (2006). A new statistical method for estimating the usual intake of episodically consumed foods with application to their distribution. J. Am. Diet Assoc..

[16] NHANES (2010). NHANES 2007–2008 Dietary Data (Dietary Interview-Individual Foods, First Day) (DR1IFF_E.). https://wwwn.cdc.gov/Nchs/Nhanes/Search/DataPage.aspx?Component=Dietary&CycleBeginYear=2007.

[17] U.S. Department of Agriculture ARS, Beltsville Human Nutrition Research Center What We Eat in America, NHANES 2009–2010, 2011–2012. https://wwwn.cdc.gov/nchs/nhanes/default.aspx.

[18] USDA Food and Nutrient Database for Dietary Studies, 5.0. 2012 October 2014. http://www.ars.usda.gov/SP2UserFiles/Place/80400530/pdf/fndds/fndds5_doc.pdf#page=64.

[19] Institute of Medicine (1998). Dietary Reference Intakes for Thiamin, Riboflavin, Niacin, Vitamin B6, Folate, Vitamin B12, Pantothenic Acid, Biotin, and Choline.

[20] Institute of Medicine (2005). Dietary Reference Intakes for Water, Potassium, Sodium, Chloride, and Sulfate.

[21] Institute of Medicine (2001). Dietary Reference Intakes for Vitamin, A., Vitamin K., Arsenic, Boron, Chromium, Copper, Iodine, Iron, Manganese, Molybdenum, Nickel, Silicon, Vanadium, and Zinc.

[22] Institute of Medicine (1997). Dietary Reference Intakes for Calcium, Phosphorus, Magnesium, Vitamin D, and Fluoride.

[23] Johnson C.L., Paulose-Ram R., Ogden C.L., Carroll M.D., Kruszon-Moran D., Dohrmann S.M., Curtin L.R. (2013). National Health and Nutrition Examination Survey: Analytic Guidelines, 1999–2010. Vital. Health Stat.

[24] CDC (2013). National Health and Nutrition Examination Survey: Analytic Guidelines, 2011–2012. https://wwwn.cdc.gov/nchs/data/nhanes/2011-2012/analyticguidelines/analytic_guidelines_11_12.pdf.

[25] CDC National Center for Health Statistics NHANES response rates. National Center for Health Statistics 2011. http://www.cdc.gov/nchs/nhanes/response_rates_CPS.htm.

[26] Kirkpatrick S.I., Dodd K.W., Reedy J., Krebs-Smith S.M. (2012). Income and race/ethnicity are associated with adherence to food-based dietary guidance among US adults and children. J. Acad. Nutr. Diet..

[27] Ross A.C., Manson J.E., Abrams S.A., Aloia J.F., Brannon P.M., Clinton S.K., Durazo-Arvizu R.A., Gallagher J.C., Gallo R.L. (2011). The 2011 report on dietary reference intakes for calcium and vitamin D from the Institute of Medicine: What clinicians need to know. J. Clin. Endocrinol. MeTable..

[28] Institute of Medicine (2000). Dietary Reference Intakes for Vitamin C, Vitamin E, Selenium, and Carotenoids.

[29] Felkner M., Suarez L., Hendricks K., Gunter E.W. (2002). Blood folate levels on the Texas-Mexico border. Tex. Med..

[30] Harley K., Eskenazi B., Block G. (2005). The association of time in the US and diet during pregnancy in low-income women of Mexican descent. Paediatr. Perinat. Epidemiol..

[31] Sharma S., Murphy S.P., Wilkens L.R., Shen L., Hankin J.H., Monroe K.R., Henderson B., Kolonel L.N. (2004). Adherence to the food guide pyramid recommendations among African Americans and Latinos: Results from the Multiethnic Cohort. J. Am. Diet Assoc..

[32] Batis C., Hernandez-Barrera L., Barquera S., Rivera J.A., Popkin B.M. (2011). Food acculturation drives dietary differences among Mexicans, Mexican Americans, and Non-Hispanic Whites. J. Nutr..

[33] Kant A.K., Graubard B.I., Kumanyika S.K. (2007). Trends in black-white differentials in dietary intakes of U.S. adults, 1971–2002. Am. J. Prev. Med..

[34] Lucan S.C., Barg F.K., Long J.A. (2010). Promoters and barriers to fruit, vegetable, and fast-food consumption among urban, low-income African Americans-a qualitative approach. Am. J. Public Health.

[35] Yeh M.C., Ickes S.B., Lowenstein L.M., Shuval K., Ammerman A.S., Farris R., Katz D.L. (2008). Understanding barriers and facilitators of fruit and vegetable consumption among a diverse multi-ethnic population in the USA. Health Promot. Int..

[36] Briefel R.R., Sempos C.T., McDowell M.A., Chien S., Alaimo K. (1997). Dietary methods research in the third National Health and Nutrition Examination Survey: Underreporting of energy intake. Am. J. Clin. Nutr..

[37] Moshfegh A.J., Rhodes D.G., Baer D.J., Murayi T., Clemens J.C., Rumpler W.V., Paul D.R., Sebastian R.S., Kuczynski K.J. (2008). The US Department of Agriculture Automated Multiple-Pass Method reduces bias in the collection of energy intakes. Am. J. Clin. Nutr..

