# Supplementary material for: Race/Ethnicity, Enrichment/Fortification, and Dietary Supplementation in the U.S. Population, NHANES 2009–2012
Source: Nutrients. 2019 May 2;11(5):1005. doi: 10.3390/nu11051005 (PMC6567168; doi:10.3390/nu11051005)
Supplement: Supplementary file 1 [file nutrients-11-01005-s001.pdf]

Table S1. Descriptive Statistics of Sample Size for Interview Day 1 and Day 2 for the U.S. Population 2 Years of Age and Older, NHANES 2009-2012

	Non-Hispanic White	Non-Hispanic Black	Hispanic	Other Race/Ethnicity*	TOTAL
**Day 1**					
PIR < 131%	1944	1571	2159	507	6181
PIR 131-185%	660	567	667	222	2116
PIR > 185%	3502	1410	1434	926	7272
Missing PIR	313	341	570	182	1406
**TOTAL**	6419	3889	4830	1837	16,975
**Day 2**					
PIR < 131%	1698	1341	1867	417	5323
PIR 131-185%	577	480	565	190	1812
PIR > 185%	3251	1257	1267	844	6599
Missing PIR	278	287	455	148	1168
**TOTAL**	5784	3365	4154	1599	14,902

U.S., United States; NHANES, National Health and Nutrition Examination Survey; PIR, Poverty Index Ratio.

* Other race includes non-Hispanic persons reporting multiple races.
